# Proprietary Preparations and the Medical Profession

**Published:** 1905-01-14

**Authors:** 


### Proprietary Preparations and the Medical Profession.

The general advantages and benefits which have been conferred on the community as a result of commercial enterprise and competition are so obvious that it is unnecessary to recite them. Whether or not in the future co-operative is to succeed competitive effort, the day of individualism, it cannot be denied, has not been an altogether inglorious one. There need be little hesitation in allowing also that in its relation to medical practice and to the health of the community the commercial spirit can count no scanty record of triumphs. The advantageous preparation and modification of many foodstuffs, the development of sanitary appliances, the advancement of the pharmaceutical forms and combinations of remedies, even improvements in methods of treatment have not invariably been due to suggestions taking origin within the ranks of the profession. On the contrary, amidst this list may be found many instances of progress due to extra-professional enterprise stimulated by a perfectly legitimate commercial ambition. Unfortunately, to the same source must be attributed many empty and vainglorious claims and much that is certainly humbug, and in some instances fraud. Still, it is well for the profession to bear in mind the possibility of good as well as of evil in the various schemes and proposals which are brought to their attention by the enterprise of business men. To the latter it is, of course, of enormous importance to secure the countenance and support of the medical profession, as is obvious in the efforts which are made to attain this end. And in some more or less definite fashion every medical man is compelled to decide the general attitude he shall adopt towards the numerous medicinal and dietetic developments which are seeking a market through the means of his support. The temperament of some leads them with sanguine expectation to adopt readily the latest proposal, whilst others invariably turn a deaf ear to everything that is new and modern. It is between these two extremes that the safe and scientific position is to be found. Whilst all this is quite true, it is necessary to remark that the medical endorsement and recommendation of a proprietary food or other preparation involves a considerable responsibility. The public attach to such testimony a high value, and, thanks to the arts of the advertiser, its influence may be a very extensive one. Especially is it necessary to be wary in dealing with preparations of unknown composition and having attractive titles. Indeed, it may be said that medical support, even in dealing with the individual patient, should only be given when it is known that the article recommended is actually what it professes to be. Under the existing state of the law names may readily be utilised so as to carry to the public mind an altogether erroneous impression of the nature of the substances which bear them, and this ambiguous use of terms in connection with foods and medicines has serious dangers. It is to be regretted that all such substances are not compelled to bear on each package an exact statement of their composition so that the purchaser would be informed what in fact he is purchasing. Take for example the term "milk powder" regarding which some correspondence appears in another column. The term is an attractive one and the preparation makes claims to many virtues. Whether all these claims are or are not well-founded is not now the question. But it is allowed that different specimens differ widely from one another, as for example in the percentage of fat. There is no definite standard or authoritative definition of this or of many other widely advertised products. It would be a great public advantage if the advertisers of proprietary foods and medicines were compelled to submit their preparations to official inspection and were liable to penalty in the event of default. In the meantime it is important that medical practitioners and hospital authorities should be careful not to lend their support to any commercial product without being fully satisfied that the actual value of this is equal to its professions. To prove all things is a sound rule of life and it is especially incumbent on those whose word is sure to enlist the suffrages of great numbers of the less trained and more gullible general public.

